# Outcome of patients with pulmonary embolism admitted to the intensive care unit

**DOI:** 10.4103/1817-1737.44779

**Published:** 2009

**Authors:** Hadeel Al Otair, Mohammed Chaudhry, Shaffi Shaikh, Ahmed BaHammam

**Affiliations:** *Respiratory Division, College of Medicine, King Saud University, Riyadh 11324, Saudi Arabia*

**Keywords:** Intensive care unit, pulmonary embolism, thrombolytic agents

## Abstract

**BACKGROUND:**

Pulmonary embolism (PE) is an important cause of in-hospital mortality. Many patients are admitted to the intensive care unit (ICU) either due to hemodynamic instability or severe hypoxemia. Few reports have addressed the outcome of patients with PE; however, none were from ICUs in the Middle East.

**OBJECTIVES:**

To describe the demographics, clinical presentation, risk factors and outcome of patients with PE admitted to the medical ICU and to identify possible factors associated with poor prognosis.

**MATERIALS AND METHODS:**

Data were collected retrospectively by reviewing the records of patients admitted to the medical ICU with primary diagnosis of PE between January 2001 and June 2007. Demographic, clinical, radiological and therapeutic data were collected on admission to ICU.

**RESULTS:**

Fifty-six patients (43% females) with PE were admitted to the ICU during the study period. Their mean age was 40.6 ± 10.6 years. Seven patients (12.5%) had massive PE with hemodynamic instability and 15 (26.8%) had submassive PE. The remaining patients were admitted due to severe hypoxemia. Recent surgery followed by obesity were the most common risk factors (55.4 and 28.6%, respectively). Four patients with massive PE received thrombolysis because the remaining three had absolute contraindications. Fatal gastrointestinal bleeding occurred in one patient post thrombolysis. Additionally, two patients with massive PE and five with submassive PE died within 72 h of admission to the ICU, resulting in an overall mortality rate of 14%. Nonsurvivors were older and had a higher prevalence of immobility and cerebrovascular diseases compared with survivors.

**CONCLUSIONS:**

The mortality rate of patients with PE admitted to the ICU in our center was comparable to other published studies. Older age, immobility as well as coexistent cerebrovascular diseases were associated with a worse outcome.

Pulmonary embolism (PE) is a major health problem with a reported annual incidence of 0.5 per 1000 persons.[1] Despite recent advances in prophylactic, diagnostic and therapeutic modalities, it is still one of the important causes of hospital morbidity and mortality.[2,3] This could be partly due to its nonspecific symptoms and scarcity of specific physical signs. Not infrequently, these patients require admission to the intensive care unit (ICU) due to hemodynamic instability or severe hypoxemia. In 2002, 101,000 patients with primary diagnosis of PE were admitted to acute care hospitals in the United States[4] with a case fatality rate of 15% at 3 months.[5] Previous reports have described the variable outcome of patients with PE with reported mortality rate ranging from 8.1% in stable patients to 25% in those with cardiogenic shock and 65% post cardiopulmonary resuscitation.[2,6] Nevertheless, there are no published studies from Middle Eastern hospitals that assessed the outcome of PE in the ICUs. Therefore, we conducted this study to determine the outcome, risk factors, clinical characteristics and demographics of patients with PE admitted to the medical ICU at King Khalid University Hospital in Riyadh, Saudi Arabia, and to identify possible demographic and clinical factors associated with poor outcome.

## Materials and Methods

Between January 2000 and June 2007, the records of all patients with primary diagnosis of PE admitted to the medical ICU in our institute were reviewed. The diagnosis of PE was confirmed by a high-probability ventilation/perfusion (V/Q) scan[7] or spiral computed tomography (CT) scan showing one or more filling defects or obstruction in the pulmonary artery or its branches.[8] Massive PE was defined as the presence of hypotension or shock whereas submassive PE was defined as stable hemodynamics in the presence of echocardiographic right ventricular(RV) dysfunction based on RV dilatation (end diastolic diameter > 30mm) or hypokinesia or abnormal movement of the interventricular septum with or without tricuspid regurgitation.[9] A data entry form was designed to collect demographic, clinical and radiological data on admission to the ICU. This included age, gender, clinical presentation, risk factors (obesity defined as body mass index > 30), immobility, recent surgery within 1 week, oral contraceptive pills and antiphospholipid syndrome), comorbid medical conditions, namely congestive cardiac failure, chronic obstructive pulmonary disease (COPD), cancer, chronic kidney diseases, connective tissue diseases and cerebrovascular diseases. Additionally, vital signs, electrocardiographic, chest X-ray findings (normal, pleural effusion, local oligemia, cardiomegaly, atelectasis, raised hemidiaphragm, prominent pulmonary artery and wedge-shaped opacity) and arterial blood gas values were recorded. Chest X-rays were read by a radiologist who was blinded to the patient's diagnosis and outcome. Therapeutic agents given, either unfractionated heparin alone or thrombolytic agent (tissue plasminogen activator, t-PA), were noted. The number of patients who died in the ICU and in the hospital was recorded as the primary clinical outcome and patients were grouped accordingly into survivors and nonsurvivors. The eight nonsurvivors were compared with 16 randomly selected survivors from 48 survivors in relation to the above-mentioned clinical variables as an unmatched case control study design.

### Statistical analysis

The data were entered in Microsoft excel and analyzed using the statistical package for social sciences (SPSS Inc., 233 S. Wacker Drive, 11^th^ Floor, Chicago, IL 60606-6307) for personal computer version 16 software. Data was expressed in text and tables as mean ± standard deviation and proportions for continuous and categorical variables. Bivariate analysis was performed using Student's *t*-test for two independent groups of the continuous variables and Fisher's exact test to observe significant association of the categorical variables in relation to the outcome. Difference was considered significant at *P* < 0.05.

## Results

Fifty-six patients (43% females) with PE were admitted to the ICU during the study period. Their mean age was 40.6 ± 10.6 years. Seven (12.5%) patients were diagnosed as massive PE and 15 (26.8%) as submassive according to the above-mentioned criteria. The remaining patients were admitted due to severe hypoxemia (mean PaO_2_ at room air = 54.89 ± 6.85 mm Hg). The diagnosis of PE was made by spiral CT in 44 patients and by V/Q scan in eight. Four patients in whom the clinical suspicion of PE was high, but were unstable to undergo any confirmative imaging, were diagnosed based on a positive duplex study of the legs showing proximal deep vein thrombosis (DVT) along with echocardiographic evidence of RV dilation and dysfunction. The clinical presentation, risk factors and comorbid conditions of the study group are shown in Table 1. Dyspnea, cough and chest pain were the main presenting symptoms in 39 (69.6%), 35 (62.5%) and 33 (58.9%) patients, respectively. Recent surgery followed by obesity were the most common risk factors in our study group (55.4 and28.6%, respectively). More than half of the patients had concomitant cardiorespiratory conditions. Chest X-ray was normal in 41 (73.2%) patients. However, the most frequent abnormality noted was cardiomegaly in 13 (23%) cases followed by lung oligemia in eight (14%) [Table 2]. Treatment was started before definitive diagnosis in the majority (87.5%) of the cases. Only four patients out of the seven who had massive PE received thrombolysis with t-PA because the remaining three patients had surgery within 72 h of the development of PE. All seven patients with massive PE required ionotropic support and were mechanically ventilated for a mean duration of 45.5 ± 12.4 h. One patient with massive PE developed fatal gastrointestinal bleeding post thrombolysis. Three other patients died within 48 h of admission to the ICU and four patients died after 72 h. Five of the patients who died had submassive and two had massive PE resulting in an overall mortality rate of 14%. Only one among those who died received thrombolytic therapy, which was given twice due to the lack of clinical improvement. Compared with survivors, nonsurvivors were found to be significantly older (56.4 ± 10.3) years vs. (46.2 ± 7.2) years, respectively (*P* = 0.01)) [Table 3]. However, there was no gender difference between the two groups. A greater number of nonsurvivors had preexisting connective tissue or cerebrovascular diseases at the time of diagnosis of PE. On the other hand, no significant difference was found in the frequency of cardiovascular, respiratory or renal conditions. All of the patients who died had recent surgery and most of them (75%) were immobile. Comparing vital signs on admission to the ICU, nonsurvivors were more tachypneic and tachycardic and their diastolic blood pressure was lower than the survivors' [Table 3]. Arterial blood gas analyses in nonsurvivors showed significantly lower PaO_2_ and O_2_ saturation; however, there was no difference in PaCO_2_ measurements.

**Table 1:** Patient characteristics at the time of admission to the medical intensive care unit

Variables	Patient (n = 56 (%))
Age (years)	40.6 (10.5)
Gender, male	32 (57)
Clinical presentation	
Dyspnea	39 (69.6)
Chest pain	33 (58.9)
Cough	35 (62.5)
Hemoptysis	14 (25)
Palpitation	22 (39.3)
Giddiness/L.O.C	6 (10.8)
Risk factors	
Obesity	16 (28.6)
Recent surgery < 72h	31(55.4)
OCP	7 (12.5)
Immobility	9 (16.1)
Concomitant diseases	
Cardiovascular	33 (58.9)
Respiratory	30 (53.6)
Diabetes	25 (44.6)
Chronic kidney disease	31 (55.4)
Connective tissue diseases	14 (25)
APLS	8 (14.3)

L.O.C. - Loss of consciousness, OCP - Oral contraceptive pills; APLS - Antiphospholipid syndrome

**Table 2:** Chest X-ray findings of all patients at the time of admission

Findings	Frequency (n = 56 (%))
Normal	41 (73.2)
Pleural effusion	5 (8.9)
Oligemia	8 (14.3)
Volume loss/atelectasis	4 (7.1)
Cardiomegaly	13 (23.2)
Prominent pulmonary artery	5 (8.9)
Wedge-shaped opacity	3 (5.4)

**Table 3:** Comparison of survivors and nonsurvivors with regard to their demographic and clinical characteristics

Variable	Nonsurvivors (n = 8), Mean ± SD	Survivors (n = 16), Mean ± SD	*P*-value
Age (years)	56.4 ± 10.3	46.3 ± 7.2	0.01
SBP	107.7 ± 19	118 ± 12.5	0.1
DBP	64.5 ± 9.8	76 ± 9.1	0.01
HR	93.5 ± 16.9	80 ± 8.19	0.015
RR	29.1 ± 8.7	23.5 ± 3	0.03
PaO_2_ (mm Hg)	47.6 ± 1.6	56.7 ± 4.6	< 0.0001
SaO_2_%	81.5 ± 4.9	87.5 ± 4.2	0.005
P_a_CO_2_ (mm Hg)	32.8 ± 4.3	34.5 ± 5.2	0.44
Comorbid condition			
Cardiovascular	100%	81.3%	0.28
Respiratory	87.5%	56.3%	0.14
Connective tissue diseases	75%	31.3%	0.05
Cerebrovascular	62.5%	6.3%	0.007
Predisposing factors			
Recent surgery	100%	43.8%	0.009
Bed ridden	75%	0%	0.0002

SBP - Systolic blood pressure, DPB - Diastolic blood pressure, HR - Heart rate, RR - Respiratory rate, pO_2_ - Partial pressure of oxygen, SaO_2_% - Oxygen saturation in arterial blood, PCO_2_ - Partial pressure of carbon dioxide

## Discussion

The outcome of patients with PE is quite variable depending primarily on the hemodynamic status and the embolus size. However, other factors have been found in many studies to be useful as prognostic indicators. In this study, we described the outcome and the factors associated with poor prognosis of patients with PE admitted to the medical ICU of a teaching hospital in Riyadh, Saudi Arabia, as no study has addressed this issue in Middle Eastern hospitals. The overall in-hospital mortality rate for the 56 patients studied in our center was 14%. The MAPPET registry reported an overall mortality rate of 1001 patients with PE to be 29% (14% in the presence of hypotension, 25% in cases of cardiogenic shock and 65% post cardiac arrest).[2] Most of the patients who died in our ICU had submassive PE and were normotensive on admission. Their sudden collapse may indicate loss of the initial cardiovascular compensatory mechanisms, mainly reflex tachycardia and systemic arterial vasoconstriction.[10] Moreover, it has been reported that 10% of the initially diagnosed submassive PE develop shock after admission with 50% mortality.[11] Early initiation of anticoagulation based on high clinical suspicion is one of the known factors contributing to a favorable outcome in such patients.[12] Older studies have shown that the rate of DVT among general surgical patients who did not receive DVT prophylaxis ranges between 15 and 30% and subsequent fatal PE between 0.2 and 0.9%[13,14] and this can further be affected by the type and duration of surgery and the type of anesthesia used.[15–17] Population-based studies estimated the risk of thrombosis to be increased two-folds in obese individuals.[18,19] Chronic lung diseases and cardiovascular diseases were the main comorbid conditions present before the diagnosis of PE in our patients. Earlier studies have identified heart failure and COPD to be the major risk factors for venous thrombo-embolism in hospitalized medical patients.[5,20,21] Aujesty and Obrosky, in their prognostic model, found these conditions to be independently associated with higher 30-day mortality indicating worse prognosis.[22]

When clinical characteristics of nonsurvivors were compared with those of survivors, older age and coexistent connective tissue and cerebrovascular diseases as well as immobility were found to be more common in the nonsurvivor group. Older age is well known to correlate poorly with survival post PE.[5,22,23] Prolonged immobilization was found to be an independent factor for increased mortality among 533 patients with PE hospitalized in Japan.[24] Nonsurvivors were significantly more tachypneic, tachycardic and hypoxemic on admission than survivors. This is in accordance with previous reports that utilized the above clinical parameters among others to stratify patients with PE into five classes with increasing mortality.[22,25,26] Although systolic blood pressure (BP) was not different among the two groups, diastolic BP on admission was significantly lower in nonsurvivors. We think this could be related to loss of the initial compensatory vasoconstriction signaling impending hypotension and shock. However, this needs to be confirmed in larger studies.

There are some limitations to this study that need to be addressed, which include the small number of patients enrolled and the retrospective nature of the study in addition to the comparison of nonsurvivors with survivors as an unmatched control group.

Cardiac biomarkers, namely Troponin and Brain natriuretic peptide, levels were not included in the comparison between survivors and nonsurvivors because they were not available for all patients.

In summary, this is the first description from a Middle Eastern country of the outcome, risk factors and clinical characteristics of patients with PE admitted to the ICU. The mortality rate in this study is comparable to previously published reports from other parts of the world, despite the fact that most of our patients had significant premorbid conditions. Older age, cerebrovascular and connective tissue diseases as well as immobility were factors associated with poor outcome.
